# Ultrathin 2D Graphitic Carbon Nitride on Metal Films: Underpotential Sodium Deposition in Adlayers for Sodium‐Ion Batteries

**DOI:** 10.1002/anie.202000314

**Published:** 2020-04-06

**Authors:** Lu Chen, Runyu Yan, Martin Oschatz, Lei Jiang, Markus Antonietti, Kai Xiao

**Affiliations:** ^1^ Key Laboratory of Bio-inspired Smart Interfacial Science and Technology of Ministry of Education School of Chemistry Beihang University 100191 Beijing P. R. China; ^2^ Max Planck Institute of Colloids and Interfaces Department of Colloid Chemistry 14476 Potsdam Germany; ^3^ Institute of Chemistry University of Potsdam Karl-Liebknecht-Straße 24–25 14476 Potsdam Germany

**Keywords:** 2D films, carbon nitride, chemical vapor deposition, sodium-ion batteries, underpotential deposition

## Abstract

Efficient and low‐cost anode materials for the sodium‐ion battery are highly desired to enable more economic energy storage. Effects on an ultrathin carbon nitride film deposited on a copper metal electrode are presented. The combination of effects show an unusually high capacity to store sodium metal. The g‐C_3_N_4_ film is as thin as 10 nm and can be fabricated by an efficient, facile, and general chemical‐vapor deposition method. A high reversible capacity of formally up to 51 Ah g^−1^ indicates that the Na is not only stored in the carbon nitride as such, but that carbon nitride activates also the metal for reversible Na‐deposition, while forming at the same time an solid electrolyte interface layer avoiding direct contact of the metallic phase with the liquid electrolyte.

## Graphical abstract

**Carbon nitride film** deposited on a copper electrode can be used to store sodium ions. The 10 nm g‐C_3_N_4_ film exhibited a high reversible capacity (up to 51 Ah g^−1^), indicating that the Na is not only stored in the carbon nitride as such, but that carbon nitride also activates the metal for reversible Na‐deposition, while forming a solid electrolyte interface layer, avoiding direct contact of the metallic phase with the liquid electrolyte.

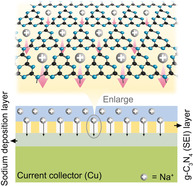

## Introduction

In view of the virtually inexhaustible and ubiquitous sodium resources around the world, sodium‐ion batteries (SIBs) are considered as an attractive alternative to lithium‐ion batteries and have received a great deal of attention in the last ten years.^1^ Anode and cathode materials, with no doubt, are the crucial factors for the successful development of a high‐performance sodium‐ion battery. A wider array of choices is available for cathodes, including high‐performance layered transition metal oxides and polyanionic compounds.^2^ The state‐of‐the‐art sodium‐ion battery cathode materials have reported performances comparable to their lithium‐ion battery counterparts. However, the anode material candidates for SIBs still lack high specific capacity and an appropriately negative redox potential at the same time.1a, 3 This is due to the fact that Li is more covalent in character and can intercalate into graphite or in intermetallic compounds, while sodium cannot. Graphite, the most commonly used anode in lithium‐ion cells, does not intercalate sodium to any appreciable extent and is electrochemically irreversible.^4^ So far, many other materials including noble carbon,^5^ Group 14 elements such as germanium, tin, or lead,^6^ and Group 15 elements such as antimony^7^ have been used as anode for SIBs, however their performances are still far from ideal values.

For carbon anode materials, the introduction of heteroatoms (such as B, N, S, and P) has been considered a promising approach to enhance the capacity for sodium battery, surface wettability, and electronic conductivity, which in turn promote the charge transfer and electrode–electrolyte interactions.^8^ So far, N‐doped carbon is the most studied heteroatom‐doped carbon material.^9^ As an analogue of graphite, graphitic carbon nitride (g‐C_3_N_4_) or the more disordered polymeric carbon nitride is composed of tri‐s‐triazine subunits connected through planar tertiary amino groups in a layer.^10^ Carbon nitrides also have a stacked two‐dimensional structure, however with countless atom‐sized pores that allow for metal incorporation and metal permeation. Graphitic carbon nitride materials have aroused much interests because of their intrinsic features, such as being a metal‐free catalyst and a polymer semiconductor.^11^ They have been used in photocatalysis,10c electrocatalysis,^12^ novel solar energy devices,^13^ ionic‐type photodetectors,^14^ and carbon nitride based actuators.^15^ Previous work has shown that even 500 nm thick layers of oriented carbon nitride are rather good ion conductors,^16^ for example, for Na^+^, while being essentially impermeable for most organic solvents. This property makes such thin carbon nitride layers ideal candidates as a solid electrolyte interface (SEI) for metal anodes in batteries. Herein, a pristine g‐C_3_N_4_ film with regular nanostructure was applied with the original intention to create such a SEI. The concept was that owing to the electric resistance of the carbon nitride film, Na^0^ is only deposited on the copper surface after permeating the g‐C_3_N_4_ film, thus the carbon nitride is moved up as an SEI layer on top of the growing sodium layer.

## Results and Discussion

**Figure 1:**
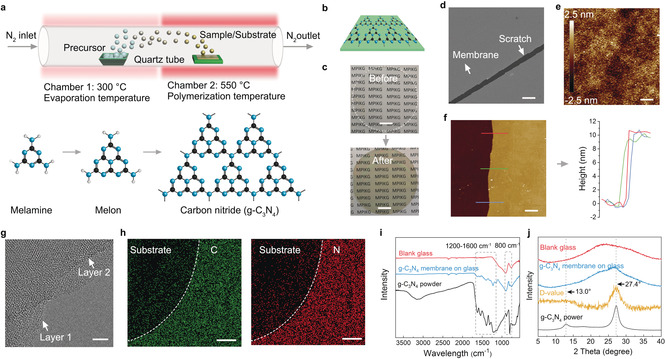
a) Illustration showing the growth of ultrathin carbon nitride film by CVD onto surfaces. b) Idealized molecular structure of the g‐C_3_N_4_ film on a substrate. c) Quartz substrate before and after film polymerization. Scale bar: 1 cm. d) SEM images of a scratch in an otherwise continuous carbon nitride film on a silicon wafer substrate. Scale bar: 10 μm. e) Mean roughness of the film as determined by AFM is about 0.51 nm. Scale bar: 1 μm. f) Typical AFM height image of the film and the corresponding height profile over a scratch showing a thickness of about 10 nm. Scale bar: 1 μm. g) High‐resolution TEM of a free standing film indicating at least local crystallinity but full orientation with layers parallel to the surface. Scale bar: 10 nm. h) EDX analysis of carbon nitride film. Scale bar: 10 μm. i) FTIR of blank glass substrate, g‐C_3_N_4_ film on glass, and g‐C_3_N_4_ powder. j) XRD of blank glass substrate, g‐C_3_N_4_ film on glass, g‐C_3_N_4_ powder, and difference value (D‐value) between g‐C_3_N_4_ film on glass and blank glass substrate.

The fabrication process was based on chemical vapor deposition (CVD) and is illustrated in Figure 1 a. In general, g‐C_3_N_4_ film can be synthesized by polymerization of appropriate precursors, such as melamine or guanidinium carbonate.^15^, ^17^ In this work, the precursor powder (melamine) is put upstream within a lower temperature zone (300 °C) sufficient for evaporation. The substrate, for example, glass, silicon, or copper is placed behind the precursor powder within the high‐temperature zone at about 550 °C for the growth of the g‐C_3_N_4_ layer. After the polymerization process, the formation of a thin film on the substrate can be already visually followed (Figure 1 b,c).

g‐C_3_N_4_ films deposited on different substrates were studied for their morphology and properties (Supporting Information, Figures S1, S2). The g‐C_3_N_4_ film was examined by scanning electron microscopy (SEM), high‐resolution transition electron microscopy (HR‐TEM), and atomic force microscopy (AFM). Figure 1 d shows the SEM image of large‐scale film with a scratch paddled by a tweezer. The film is smooth and has no obvious defects even down to the nanoscale. The mean roughness of the film is about 0.51 nm (Figure 1 e). The thickness of the film used in this work is around 10 nm, which can be imaged taking an AFM height profile across the scratch on the film deposited on the Si wafer (Figure 1 f). Meanwhile, the film is at least nanocrystalline in the plane and has a rather uniform orientation of the structure.^11^ HR‐TEM imaging demonstrates the remarkably ordered interlayer stacking, confirming the high crystallinity of the film (Figure 1 g).

The formation of g‐C_3_N_4_ film can also be confirmed by energy‐dispersive X‐ray (EDX) spectroscopy (Figure 1 h). Also, the chemical composition of the film is spot on g‐C_3_N_4_ (Supporting Information, Figures S3, S4). The stretching modes of CN heterocycles from 1200 to 1600 cm^−1^ and the characteristic breathing mode of the heptazine units at 800 cm^−1^ are also found in FTIR spectra (Figure 1 i). Moreover, the peak found in gracing incidence illumination corresponding to the (002) plane of the structure clearly confirms its layered structure parallel to the plane (Figure 1 j). X‐ray photoelectron spectroscopy (XPS) also showed the typical C 1s and N 1s peaks, consistent with previous work (Supporting Information, Figure S5). All of these results provide evidence for the formation of the g‐C_3_N_4_ structure as a top coating layer.^18^

The electrochemical performance of the g‐C_3_N_4_ film deposited on a Cu foil was then evaluated directly as a flexible electrode for sodium ion batteries. To our deep surprise, Na^0^ deposition started well below the standard reduction potential of Na and continued for a rather high charge capacity, that is, contrary to standard electrodes where a slight overpotential to nucleate Na is needed,^19^ we observed pronounced underpotential deposition.^20^ This is why we decided to use and analyze the sodium deposition only in this underpotential mode. This is of course an academic mode case, but can be considered as safe and more controlled, as metallic sodium at other positions cannot form thermodynamically under these conditions at all.

**Figure 2:**
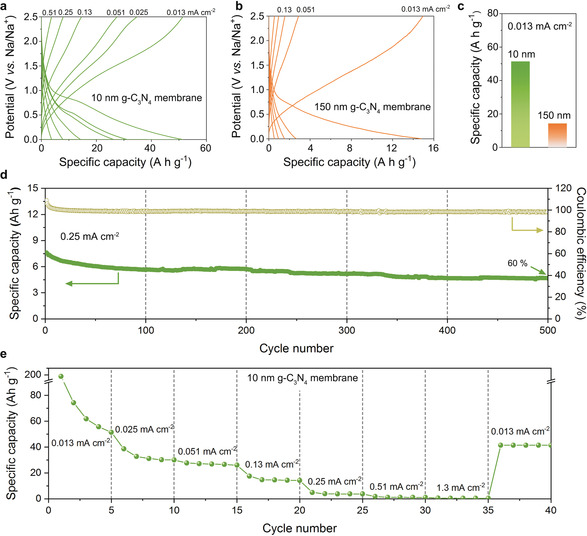
a) Galvanostatic profiles of 10 nm thick g‐C_3_N_4_ film at various areal current densities (0.013, 0.025, 0.051, 0.13, 0.25, 0.51 mA cm^−2^). b) Galvanostatic profiles of 150 nm thick g‐C_3_N_4_ film at various areal current densities (0.013, 0.051, 0.13, 0.25, 0.51 mA cm^−2^). Note: The capacity is calculated based on the film weight only. c) Comparison of the apparent capacities of 10 nm and 150 nm g‐C_3_N_4_ film on Cu foil at current density of 0.013 mA cm^−2^. d) Long‐term cycling discharge capacities and coulombic efficiencies of 10 nm carbon nitride film at 0.25 mA cm^−2^. e) Rate performance of the film at various areal current densities.

The 10 nm g‐C_3_N_4_ film showed a high capacity of 0.036 mAh at an areal current density of 0.013 mA cm^−2^. Considering the theoretical weight of 10 nm thick film of about 0.7 μg, the specific capacity of the film is calculated to be 51 Ah g^−1^ (Figure 2 a), which is unphysically high compared to the state‐of‐the‐art sodium storage materials (usually lower than 0.1 Ah g^−1^) and even 40 times higher than metallic sodium with 1.16 Ah g^−1^.1b, 19 As a reference, the bare Cu foil electrode exhibits a very low reversible capacity of 0.006 mAh at the same areal current density (Supporting Information, Figure S6), indicating that the Cu current collector alone is not responsible for this high capacity, and that the increase mostly originates (directly or indirectly) from the g‐C_3_N_4_ film. The increase of the film thickness to 150 nm results in an increase of capacity to 0.148 mAh, while the apparent specific capacity dropped to 14 Ah g^−1^ (Figure 2 b,c), which shows that the effect also works for thicker films, but seems not to be directly related to the carbon nitride as such. In fact, the thicker foil stores a higher electric energy, but division by the film weight lowers the apparent specific capacity.

The 10 nm g‐C_3_N_4_ film electrode shows a good coulombic efficiency up to 98 % and good cycling stability after 500 cycles at 0.25 mA cm^−2^ (Figure 2 d) with a capacity retention of 60 %. The rate capability of the thin g‐C_3_N_4_ film is however weak (Figure 2 e): the capacity almost vanishes even at a current density of 0.51 mA cm^−2^, indicating that the rate performance is indeed restricted by the still insufficient Na^+^ transport into or through the carbon nitride film. Recalculated the areal current to a sodium permeability, we calculate for the minimal current 780 Na^+^ ions per nm^2^ per second or about twice this number per channel, if all channels are active and accessible. This is for molecular channels of 10 nm length already a comparably high transport rate.

**Figure 3:**
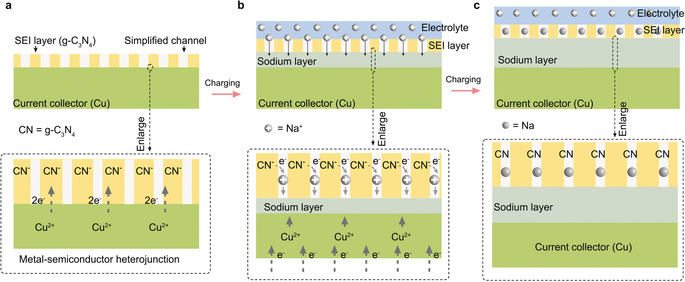
Illustration of the mechanism of the sodium storage in g‐C_3_N_4_ film activated Cu foil. a) The g‐C_3_N_4_ deposited on Cu foil (current collector) constitutes a metal–semiconductor heterojunction in which electrons will transfer from copper to g‐C_3_N_4_. b) In the charging process, Na^+^ ions permeate through thin g‐C_3_N_4_ films and deposit onto the Cu metal by underpotential deposition, thus forming there a Cu‐Na compound layer as sodium metal anode. c) The state after charging: sodium metal acts as the anode material and g‐C_3_N_4_ film works as SEI to avoid direct contact of the metallic sodium and liquid electrolyte.

The remarkable apparent specific capacity can be attributed to the specific characteristics of g‐C_3_N_4_. Previous theoretic simulations and experimental observations have demonstrated that carbon nitride itself as the anode material only has a low capacity because of the irreversible sodium uptake process and pore electrical conductivity,4b while the ultrathin 2D carbon nitride film deposited on a copper metal electrode used here has a different mechanism. Our previous work has shown that thin g‐C_3_N_4_ films have a porous structure which is permeable for Na^+^ ions at least in water,^16^ and has a good electrolyte wettability (Supporting Information, Figure S7), so it could work as a solid electrolyte interphase (SEI) layer for Na^+^ ions transport (Figure 3). The Na^+^ ions can now be reduced and deposit onto the Cu metal, thus forming there a sodium layer of in principle infinite thickness, which would constitute a protected sodium metal anode with SEI protection.

The point to address is the reason for underpotential deposition.20a, 21 By dividing the areal capacity by the molecular capacity of Na, we can calculate a Na‐film thickness of about 500 nm to be responsible for such a charge uptake. This increases for the thicker film even to about 2 μm, indicating that this is an effect related to the volume of the carbon nitride layer. It is well known that carbon nitride forms semiconductor‐metal heterojunctions (Figure 3 a), described for diverse metals, and that its HOMO position or work function is around +1.5 V.^22^ This is to be compared to Cu with + 0.34 V, that is, in the very first contact zone we expect due to the Schottky layer an electron depletion in Cu of about 1.2 V. This nicely goes with the starting underpotential of 1.2 V found for the Na^0^ deposition, that is, sodium is preferentially inserted in this layer to give back the copper its electrons, or in another view, Na^0^ in this layer can form at lower potentials, as there is energy gained by the back‐transfer of electrons to the Schottky zone of the copper (Figure 3 b). This electron back donation of course saturates and becomes less and less with increasing sodium layer thickness, and obviously at 500 nm thickness the effect of the heterojunction has vanished (Figure 3 c). The fact that this layer thickness depends on the thickness of the carbon nitride film reflects the physical transfer of electrons into the carbon nitride layer and a maximal number of electrons into the carbon nitride bulk due to structural reasons. Note that the coulomb charge in carbon nitride is presumably counterbalanced by Na^+^ in the pores, but that carbon nitride structurally only can charge up to one charge per 2 heptazine units, as shown in chemical reduction experiments.^23^

Sodium is thereby deposited in a very controlled area of the heterojunction material and in a very special electronic zone in the interlayer, which furthermore makes deposition locally very controlled under the SEI layer and excludes dendrite formation or uncontrolled Na‐deposition for thermodynamic reasons. The structure of this sodium adlayer is still unclear and deserves further structural examination, for example, by NMR. Even partial alloying with Cu cannot be excluded, as this metal is in a special, electron poor thermodynamic state, not comparable to ordinary metallic sodium.

**Figure 4:**
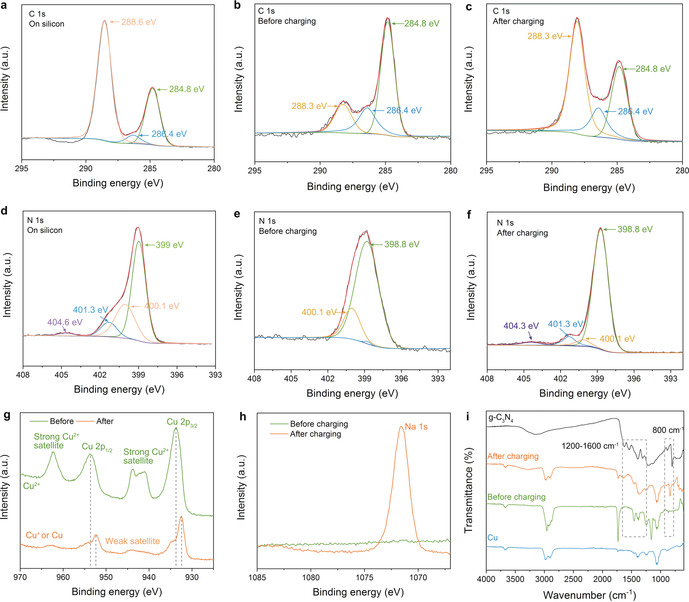
a)–h) XPS analysis of g‐C_3_N_4_ on silicon and on copper before and after charging. a) C 1s on silicon, b) C 1s on copper (before charging), c) C 1s on copper (after charging), d) N 1s on silicon, e) N 1s on copper (before charging), f) N 1s on copper (after charging), g) Cu 2p on copper before and after charging, h) Na 1s on copper before and after charging. i) Infrared spectra of g‐C_3_N_4_ power, Cu foil and g‐C_3_N_4_ film on Cu foil before and after charging.

X‐ray photoelectron spectroscopy (XPS) indeed gives evidence to follow the mechanism of sodium storage on a local scale (Figure 4; Supporting Information, Figure S8). First, the C 1s and N 1s spectra between the g‐C_3_N_4_ film on silicon and on copper (before charging) are different and quantitatively confirm the generation of a g‐C_3_N_4_‐Cu heterojunction.22d, 24 On silicon substrates, the C 1s spectrum of g‐C_3_N_4_ film can be fitted using three peaks with binding energies of 288.6 eV, 286.4 eV, and 284.8 eV (Figure 4 a), the typical peaks of g‐C_3_N_4_.^18^ On copper substrate (before charging), the high energy peak of 288.6 eV decreased to 288.3 eV and the peak area decreased obviously (Figure 4 b), which means electrons transformation from copper to g‐C_3_N_4_ film in the heterojunction. The N 1s spectrum of g‐C_3_N_4_ film on copper (before charging) however only has two peaks of 400.1 eV and 398.8 eV compared to g‐C_3_N_4_ film deposited on silicon which has four peaks (Figure 4 d,e). The disappearance of peaks in 404.6 eV and 401.3 eV (Figure 4 e) quantifies also electron transfer from copper to g‐C_3_N_4_ film to the most positive nitrogen atoms, also seen in the copper spectra (Figure 4 g). After charging, the C 1s spectra completely invert peak intensities (Figure 4 c) while the disappeared two peaks of N 1s spectra are back (Figure 4 f), both the C 1s and N 1s spectra on copper after charging are comparable to the one on silicon substrate (Figure 4 a,d). This phenomenon shows that carbon nitride layer moved up with the growing sodium layer and the effect of semiconductor‐metal heterojunction disappeared. Furthermore, the difference of the peaks is about 3 V and corresponds to the outer voltage applied while loading, that is, the carbon nitride film contains more electrons, localized at the electron poor carbon atoms as expected from the relative orbital composition of the lower boarder of the conduction band. This extra charge is compensated by a Na^+^ species (Figure 4 h), which obviously is now contained in the g‐C_3_N_4_ channels to establish electroneutrality.

The 10 nm SEI film is thin enough to see also the copper signals through the film, which allows to complete the electron flux diagram also on the copper side. Before loading, the Cu spectrum is similar to a Cu^2+^ species at the very top of the film, which could be partially surface oxidation, but also has to reflect the 1.5 V heterojunction to the carbon nitride. After loading, the Cu is still electron poor, but now more like a Cu^+^ species, with the missing charge for balance taken up by the incorporation of Na^+^ within the carbon nitride film (Figure 4 g). Meanwhile, the FTIR spectra (Figure 4 i) and SEM after charging (Supporting Information, Figure S9) also confirms the same conclusion. The slight difference between before charging and after charging reflects electron transfer, while typical stretching modes of CN heterocycles from 1200 to 1600 cm^−1^ and the characteristic breathing mode of the heptazine units at 800 cm^−1^ after charging support that the structure of the g‐C_3_N_4_ film stays structurally unchanged.

Despite such a high specific capacity, we have to underline that the present set‐up is not effective for real energy storage but rather illustrates only the fundamental effect of storing Na in adlayers between a metal and a porous semiconductor activation film. With a zone of about 500 nm thickness where Na underpotential deposition is effective, the metal conductor should be of the order of 100 nm thickness to make the whole device in the view of an engineered system storage‐efficient. Another option is to extend the charging potential and leave the underpotential deposition range. In any case, the rate of sodium permeation through carbon nitride is still too low for real devices, a problem which in turn should be handled either by smaller, more solvating solvents or by similar SEI materials with wider pores, such as PHIs,^25^ or even COFs.^26^

## Conclusion

Ultrathin and ordered carbon nitride films were grown on metallic substrates, here copper, via an easy, efficient, and facile deposition method. We then used the known ion permeability of carbon nitride films to employ them as a SEI material for a sodium battery anode. A 10 nm thick carbon nitride film deposited on Cu foil substrate was directly evaluated as a flexible electrode. When calculated to the weight of carbon nitride alone it showed an unphysically specific capacity of 51 Ah g^−1^, rather indicting the formation of a ca 500 nm thick metallic sodium layer below the carbon nitride film, deposited under underpotential conditions. This adlayer increases to about 2 μm when applying a 150 nm carbon nitride film, then however with an inferior rate behavior. It was reasoned and proven by detailed XPS experiments that this underpotential deposition is enabled by the metal–semiconductor heterojunction between copper and carbon nitride and the very positive work function of carbon nitride, creating an electron poor copper which stabilized the metallic sodium in a communicating electronic system. This effect is then restricted by the chemical capacity of carbon nitride to take up interface electrons, the molecular base for the extension of the altered interface potentials.

Despite the unexpected large size of this interface layer, its use in a practical device is limited by two‐dimensional character of the here employed electrode and the still‐too‐slow sodium permeation through the carbon nitride film. We however believe that this work opens up a general concept of using synthetic covalent SEI layers with controlled porosity and electronic properties to integrate into the next generation of batteries concepts, playing there an important role to improve stability and safety but also current capacity restrictions.

## Conflict of interest

The authors declare no conflict of interest.

## Supporting information

As a service to our authors and readers, this journal provides supporting information supplied by the authors. Such materials are peer reviewed and may be re‐organized for online delivery, but are not copy‐edited or typeset. Technical support issues arising from supporting information (other than missing files) should be addressed to the authors.

Supplementary
