# Supplementary material for: Ultrathin 2D Graphitic Carbon Nitride on Metal Films: Underpotential Sodium Deposition in Adlayers for Sodium‐Ion Batteries
Source: Angew Chem Int Ed Engl. 2020 Apr 6;59(23):9067–73. doi: 10.1002/anie.202000314 (PMC7317766; doi:10.1002/anie.202000314)
Supplement: Supplementary file 1 — Supplementary [file ANIE-59-9067-s001.pdf]

Angewandte Chemie
Eine Zeitschrift der Gesellschaft Deutscher Chemiker

Supporting Information

Ultrathin 2D Graphitic Carbon Nitride on Metal Films: Underpotential Sodium Deposition in Adlayers for Sodium-Ion Batteries

*Lu Chen, Runyu Yan, Martin Oschatz, Lei Jiang, Markus Antonietti, and Kai Xiao**

anie_202000314_sm_miscellaneous_information.pdf

Materials. Unless otherwise noted, all of the commercial reagents were used as received. Melamine, sodium perchlorate, ethylene carbonate, propylene carbonate and fluoroethylene carbonate were purchased from Sigma-Aldrich. Silicon wafers/Cu foil/glass substrates were purchased from commercial company. Before CVD, the substrates were cut into a designed size and treated with plasma or acid etching.

Characterizations. X-ray photoelectron spectroscopy (XPS) was performed by an ESCALab220i-XL electron spectrometer from VG Scientific using 300W Al Kα radiation, and the base pressure was about 3×10^{-9} mbar. The binding energies were referenced to the C1s line at 284.8 eV from adventitious carbon. For the g-C_3N_4 film on silicon and copper before charging, the samples are measured directly after deposition of film. For the g-C_3N_4 film on copper after charging, the sample is measured after one cycle charging process. The scanning electron microscope (SEM) JSM-7500F (JEOL) at an accelerating voltage of 3 kV was used to get the top view and cross section of the film. The TEM is a double-corrected Jeol ARM200F, equipped with a cold field emission gun. The acceleration voltage was set to 200kV and the emission was put to 10 μA. Electron energy loss spectra (EELS) is equipped with a Gatan GIF Quantum for energy-loss measurements. X-ray diffraction (XRD) patterns were recorded with a Bruker D8 Advance instrument with Cu Kα radiation. Fourier transform infrared (FT-IR) spectra were recorded with a Thermo Scientific Nicolet iS5 FT-IR spectra meter.

Fabrication of ultrathin carbon nitride film. The fabrication process of g-C_3N_4 film is realized by CVD. Typically, melamine (2-3g for 10 nm, 20 g for 150 nm) was put upstream within the low-temperature zone (300 °C) for evaporation. The substrate, e.g. glass, silicon, and cooper is placed behind the precursor powder within the high-temperature zone about 550 °C for the growth of g-C_3N_4. In the whole process, nitrogen is used to protect the system from oxygen and carry precursor vapor from the upstream of the tube during the reaction. After the temperature naturally cooled down to room temperature, the deposition of a transparent film on the substrate can be obtained. At last, the ultra-thin g-C_3N_4 film deposited on different substrates was used for further measurement and sodium-ion battery applications.

Electrochemical measurements. The g-C_3N_4 films grown on Cu foil with a diameter of 10 mm were directly used as working electrodes. The half-cell tests were carried out using Swagelok-type cells assembled in an argon filled glove box (H_2O < 0.1 ppm, O_2 < 0.1 ppm). Sodium metal (11 mm in diameter) was used as both counter and reference electrodes. Glass fibers (Whatman GF/C, 13 mm in diameter) were employed as the separators. The electrolyte was 1 M $NaClO_4$ in ethylene carbonate/propylene carbonate/fluoroethylene carbonate (45:45:10 by mass). The Swagelok cell was assembled using the working and counter electrodes sandwiching the separator, with 300 μL

electrolyte. A Biologic MPG-2 galvanostat/potentiostat was used for electrochemical characterization. All measurements were performed at room temperature. Cyclic voltammetry (CV) tests were performed at scan rates of 0.1 mV s^{-1}. Galvanostatic charge/discharge with potential limitation (GCPL) was applied at areal current densities between 0.013 and 1.3 mA cm^{-2} in a voltage range from 0 to +2.5 V. The specific capacity, Q (Ah g^{-1}), was calculated according to the following equation:

$$Q = \frac{Q_{dis}}{m} \quad (1)$$

Where Q_{dis} (Ah) is the charge of the discharging cycle, and m (g) is the mass of the corresponding g-C_3N_4 film.

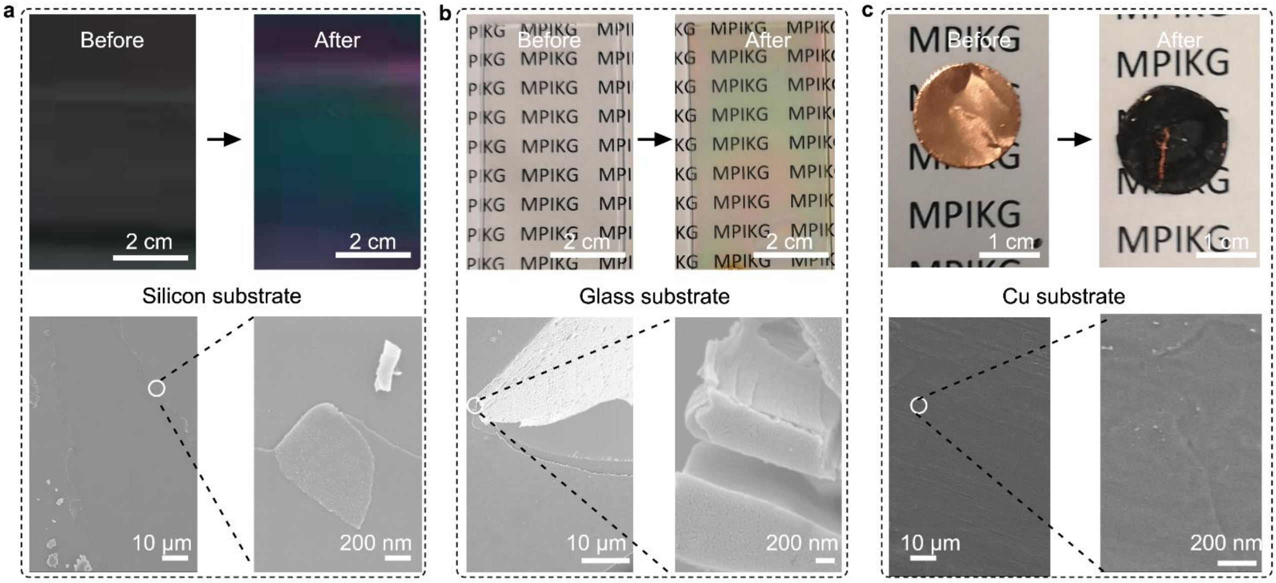

Figure S1. Photograph and SEM images of silicon substrate (a), glass substrate (b), and Cu foil (c) substrates before and after g-C_3N_4 film polymerization.

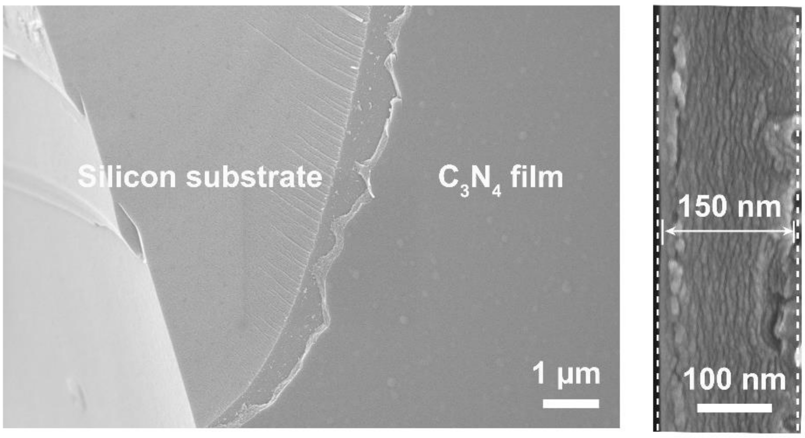

Figure S2. SEM images of 150 nm thick g-C_3N_4 film at silicon substrate.

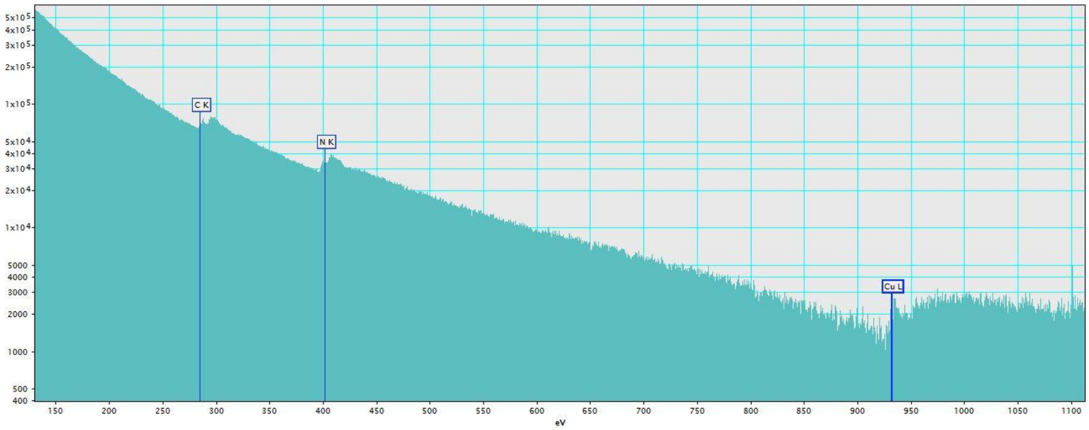

Figure S3. Electron energy loss spectroscopy of g-C_3N_4 film deposited on Cu foil showed the C/N ratio.

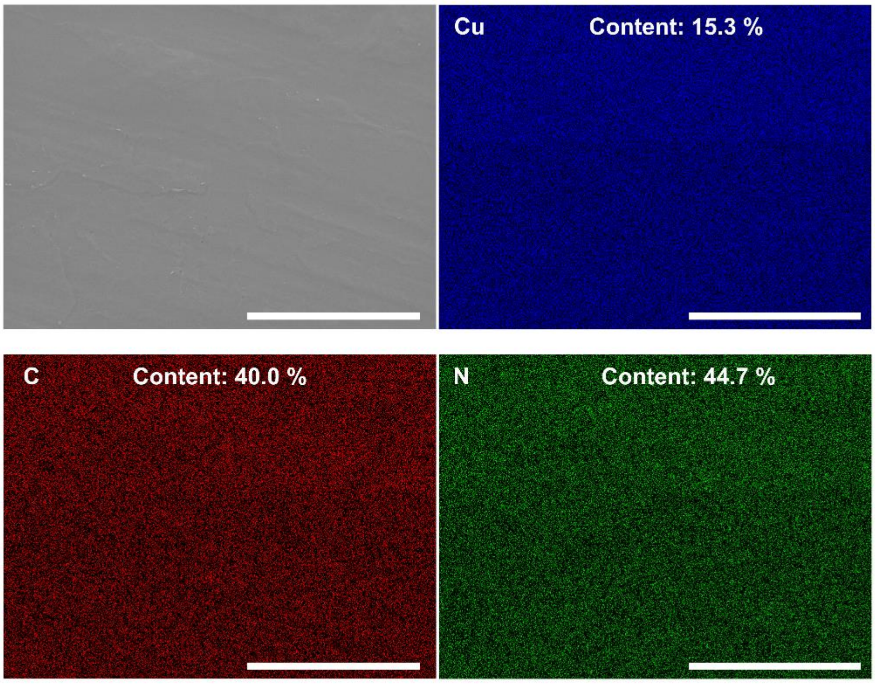

Figure S4. SEM of g-C_3N_4 film deposited on Cu foil substrate and the element map, scale bar 10 µm.

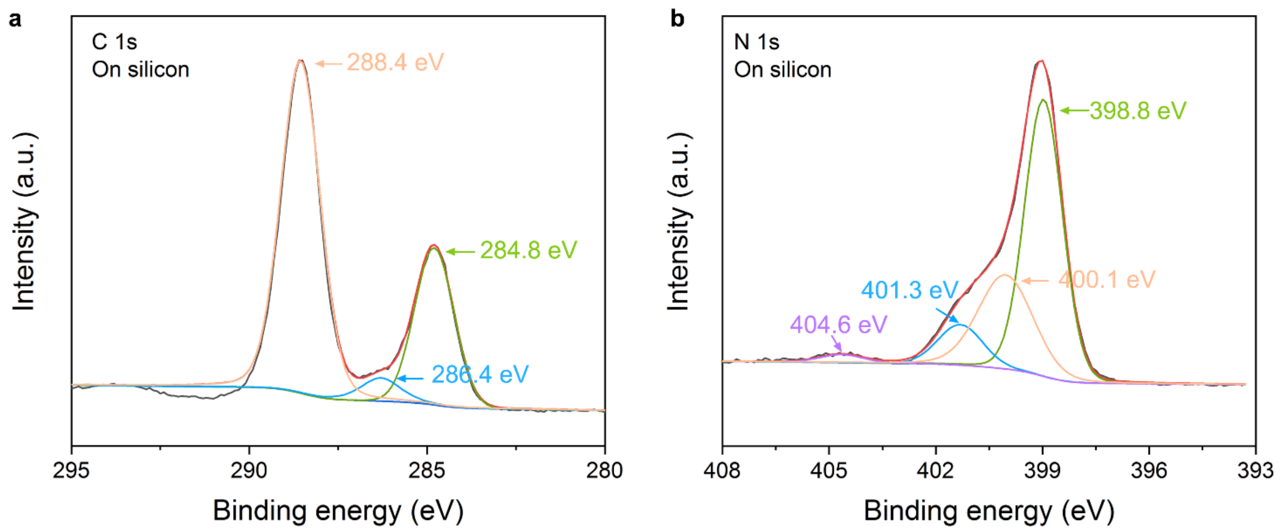

Figure S5. High-resolution C 1s (a) and N 1s (b) XPS spectra of g-C_3N_4 film.

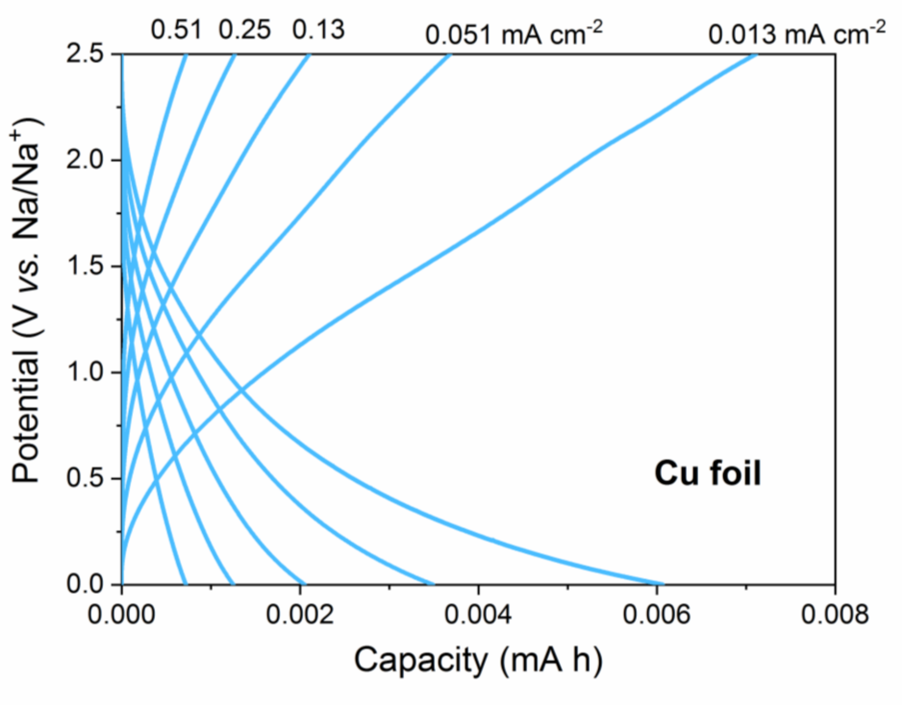

Figure S6. Galvanostatic profiles of Cu foil at various areal current densities (0.013, 0.025, 0.051, 0.13, 0.25, 0.51 mA cm^{-2}).

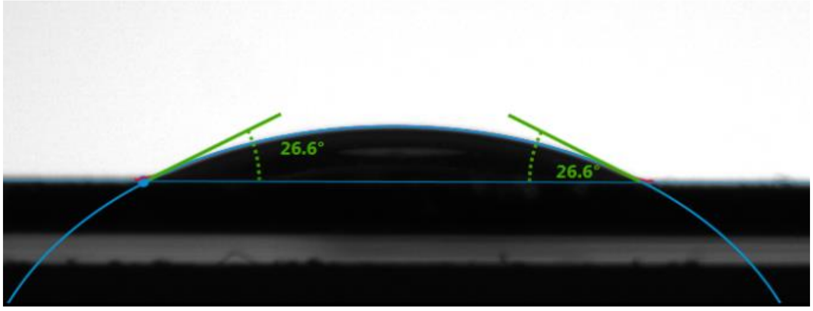

Figure S7. The electrolyte wettability of g-C_3N_4 film, with contact angle of about 26 º.

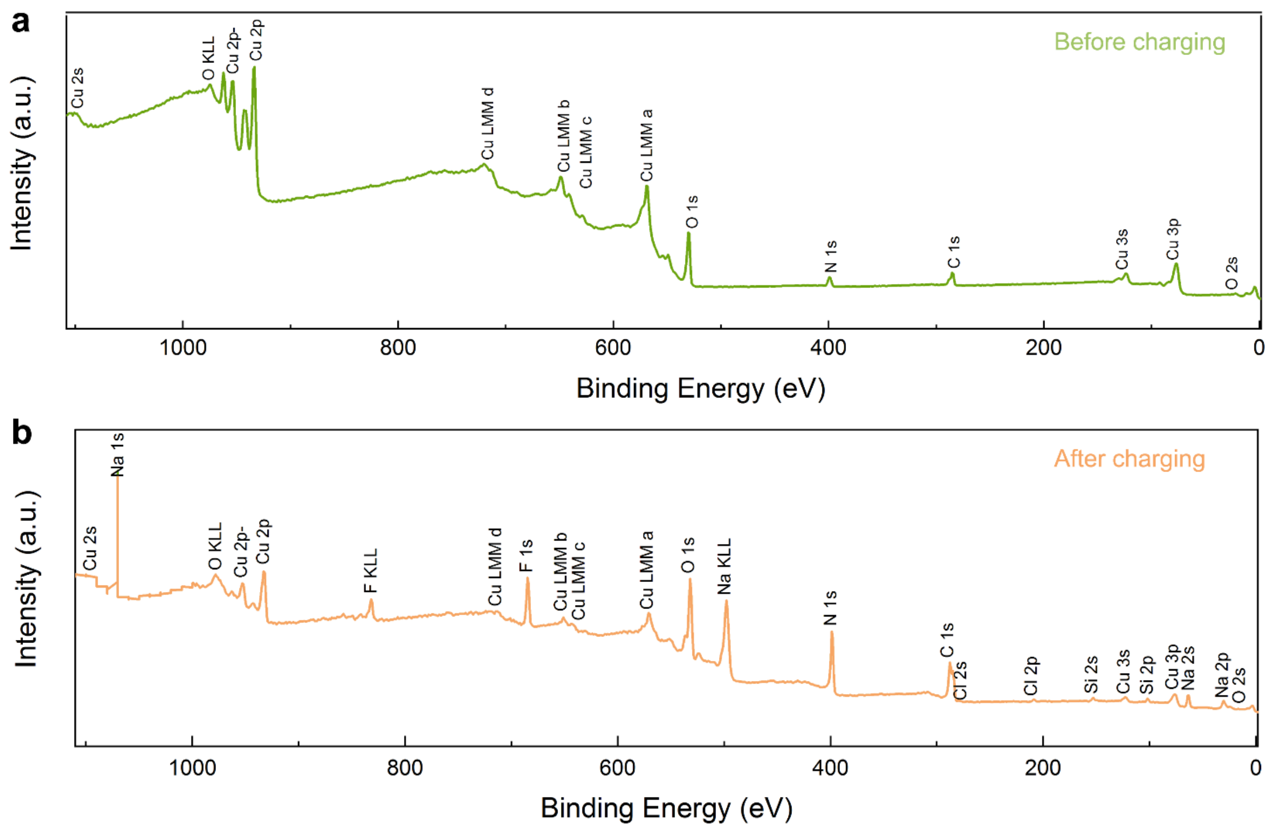

Figure S8. XPS spectra of g-C_3N_4 film @ Cu foil before (a) and after charging (b).

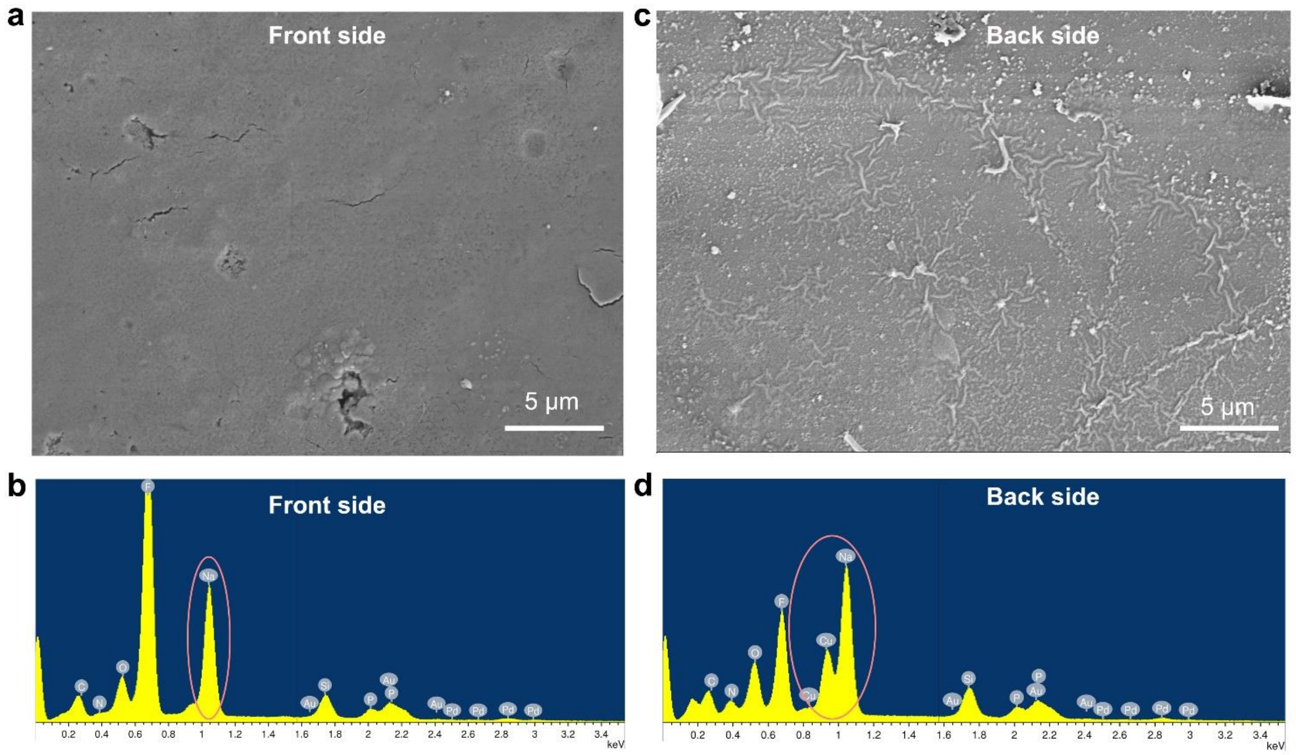

Figure S9. (a and b) SEM image and EDX spectroscopy of the front side after charging. (c and d) SEM image and EDX spectroscopy of the back side after charging. In the front side, the membrane surface is still smooth (a), and there is almost no Cu signal while strong Na signal because of the remnant of electrolyte (b). In the back side contacting with Cu collector, SEM image shows obvious salt substance (c), maybe resulting from sodium (or its derivative). Meanwhile, there is both strong Cu signal and strong Na signal (d), which means Na ions transport across the C_3N_4 film and deposited on the surface.
